# Cyclooxygenases Inhibitors Efficiently Induce Cardiomyogenesis in Human Pluripotent Stem Cells

**DOI:** 10.3390/cells9030554

**Published:** 2020-02-27

**Authors:** Harshal Nemade, Aviseka Acharya, Umesh Chaudhari, Erastus Nembo, Filomain Nguemo, Nicole Riet, Hinrich Abken, Jürgen Hescheler, Symeon Papadopoulos, Agapios Sachinidis

**Affiliations:** 1 Institute of Neurophysiology, Faculty of Medicine, University of Cologne, Robert-Koch-Str. 39, 50931 Cologne, Germany; hnemade@uni-koeln.de (H.N.); aacharya@uni-koeln.de (A.A.); umeshchaudhari80@gmail.com (U.C.); erastus.nembo@uni-koeln.de (E.N.); filo.nguemo@uni-koeln.de (F.N.); j.hescheler@uni-koeln.de (J.H.); symeon.papadopoulos@uk-koeln.de (S.P.); 2 Department I Internal Medicine and Center for Molecular Medicine Cologne (CMMC), University of Cologne (UKK), Robert-Koch-Str. 21, 50931 Cologne, Germany; nicole.riet@uk-koeln.de; 3 Regensburg Centre for Interventional Immunology (RCI), Deptartment Genetic Immunotherapy, University Hospital Regensburg, 93053 Regensburg, Germany; Hinrich.Abken@klinik.uni-regensburg.de; 4 Center for Molecular Medicine Cologne (CMMC), University of Cologne, Robert-Koch-Str. 21, 50931 Cologne, Germany

**Keywords:** Pluripotent stem cells, Cardiomyocytes, Differentiation, Sulindac, Small molecules, Wnt pathway, Cyclooxygenase pathway

## Abstract

Application of human pluripotent stem cell-derived cardiomyocytes (hPSC-CMs) is limited by the challenges in their efficient differentiation. Recently, the Wingless (Wnt) signaling pathway has emerged as the key regulator of cardiomyogenesis. In this study, we evaluated the effects of cyclooxygenase inhibitors on cardiac differentiation of hPSCs. Cardiac differentiation was performed by adherent monolayer based method using 4 hPSC lines (HES3, H9, IMR90, and ES4SKIN). The efficiency of cardiac differentiation was evaluated by flow cytometry and RT-qPCR. Generated hPSC-CMs were characterised using immunocytochemistry, electrophysiology, electron microscopy, and calcium transient measurements. Our data show that the COX inhibitors Sulindac and Diclofenac in combination with CHIR99021 (GSK-3 inhibitor) efficiently induce cardiac differentiation of hPSCs. In addition, inhibition of COX using siRNAs targeted towards COX-1 and/or COX-2 showed that inhibition of COX-2 alone or COX-1 and COX-2 in combination induce cardiomyogenesis in hPSCs within 12 days. Using IMR90-Wnt reporter line, we showed that inhibition of COX-2 led to downregulation of Wnt signalling activity in hPSCs. In conclusion, this study demonstrates that COX inhibition efficiently induced cardiogenesis via modulation of COX and Wnt pathway and the generated cardiomyocytes express cardiac-specific structural markers as well as exhibit typical calcium transients and action potentials. These cardiomyocytes also responded to cardiotoxicants and can be relevant as an in vitro cardiotoxicity screening model.

## 1. Introduction

Generation of functional cardiomyocytes (CMs) from human pluripotent stem cells (hPSCs) including, human embryonic stem cells (hESCs) and human-induced pluripotent stem cells (hiPSCs), offer a relevant tool not only to study the cardiovascular development, but also for disease modelling, toxicity studies, and drug screening [1]. However, for an in vitro cardiotoxicity screening, it is controversially discussed whether both hESCs and hiPSC-derived CMs are equally suitable [2,3,4,5,6,7]. Recent advancements in differentiation techniques allowed the generation of functional CMs from hPSCs, demonstrated by their ability to express CM-specific markers that are considered as reliable and renewable cellular source for cardiac regeneration [1,8,9]. Various signaling pathways interact in a complex and sequential manner to drive cardiomyogenesis during development [10]. Among the soluble signaling pathways, Wingless (Wnt) signaling pathway and transforming growth factor β (TGFβ) pathway have emerged as the key regulators of cardiogenesis [11,12]. Temporal modulation of Wnt/beta-catenin signaling using Wnt ligands like Wnt-C59, XAV939, KY0211, IWP2, and IWR-1 has shown to be essential and sufficient for efficient cardiac induction in hPSCs under defined, growth factor-free conditions [1,13,14]. Another challenge of hPSCs-derived CMs is the tumorigenic potential of undifferentiated cells which also raises safety concerns for the use in clinical applications [15,16,17,18].

We screened 10 small molecule Wnt inhibitors (IWP2, Wnt-C59, XAV939, IWR1endo, KY02111, Sulindac, FH535, 3289-8625, PKF118-310 and PNU-74654) using human HES3-NKX2-5^eGFP/w^ (HES3) cells [19] for their cardiogenic potential. The quantification of *NKX2-5* driven eGFP expression and spontaneous beating was used to monitor the cardiac differentiation. Sulindac at 10 µM found to be the most effective cardiogenic agent.

Previous studies have shown that Sulindac not only inhibit Wnt signaling but also inhibits cyclooxygenase-1 (COX-1) and cyclooxygenase-2 (COX-2) and is well studied for its anti-inflammatory and antineoplastic potential [20,21]. This suggests that COX-1 and COX-2 inhibition by Sulindac might also play an important role in cardiogenesis. Therefore, first we investigated the effects of Sulindac on the cardiomyogenesis in four hPSC lines and second, to ascertain the role of COX-1 and COX-2 in cardiogenesis, we knocked down COX-1 and COX-2 expression in hPSCs either by introducing siRNAs targeted towards COX-1 and/or COX-2 or by treatment with different non-steroidal anti-inflammatory drugs (NSAIDs) (e.g., Piroxicam: COX-1 inhibitor, Nimesulide: COX-2 inhibitor and Diclofenac: Non-selective COX-1 and COX-2 inhibitor). We observed generation of spontaneously beating clusters in hPSCs treated with NSAIDs and siRNAs. Inhibition of COX-2 alone and COX-1 and COX-2 together resulted in maximum number of CMs whereas inhibition of only COX-1 showed no significant increase in numbers on CMs. Further fluorescence analysis showed that inhibition of COX-1/2 results in reduced TCF-LEF promoter activity suggesting reduced Wnt signaling. These findings demonstrate for the first time that (1) Sulindac and other NSAIDs can efficiently differentiate hPSCs into functional CMs with high yields, (2) selective, stage-specific inhibition of COX-1 and COX-2 promote cardiac differentiation, (3) Wnt signaling and COX pathway both are collectively involved in cardiomyogenesis and (4) inhibition of COX leads to downregulation of WNT signalling in stem cells.

## 2. Materials and Methods

### 2.1. Maintenance of HES3-NKX2-5^eGFP/w^ (HES3) and hPSC Cells

Importation of the HES3 and subsequent experiments using hPSCs were authorised by the Robert-Koch Institute (Berlin, Germany) under license number AZ 3.04.0210083. The hPSCs were maintained as undifferentiated colonies on Corning^®^ Matrigel^®^ hESC-Qualified Matrix (Corning GmbH, Kaiserslautern, Germany) coated plates in StemMACS™ iPS-Brew XF media (Milteny Biotech, Bergish Gladbach, Germany) supplemented with 50 U/mL penicillin, and 50 U/mL streptomycin (Thermo Fisher, Waltham, MA, USA) at 37 °C and 5% CO_2_. Medium was changed every other day. When confluent, the hPSC colonies were dissociated into single cells using StemPro^®^ Accutase^®^ Cell Dissociation Reagent (Thermo Fisher, Waltham, MA, USA) and plated onto Matrigel-coated 60mm plates (Corning GmbH, Kaiserlautern, Germany).

### 2.2. Chemicals

All small molecule WNT inhibitors and NSAIDs were purchased from Tocris Bioscience, Bristow, UK. Stock solutions of 10 mM were made (in DMSO) and stored as small volume aliquots in tightly sealed sterile tubes at −20 °C. Drug dilutions were performed in pre-warmed (37 °C) RPMI medium (Gibco) supplemented with B-27 without insulin (RPMI/B-27^-ins^).

### 2.3. Cardiac Induction in Monolayer Culture

Undifferentiated HES3 cells were dissociated and seeded on matrigel-coated 60 mm plates at 3 × 10^5^ cells/plate and maintained in iPS-Brew XF media with media changed on every alternate day. When cells achieved desired confluence (≥80%), cardiac differentiation was induced by adding CHIR99021 (10 µM) in RPMI/B-27^-ins^ media (day 0 to day 1). The medium was then changed to basal RPMI/B-27^-ins^ medium and cells were kept for further 24 h. At day 2, RPMI/B-27^-ins^ medium with small molecule WNT inhibitor (2.5 µM, 5 µM and 10 µM) was added and cells were kept for 48 h (day 2 to day 4). Afterwards, cells were maintained in basal RPMI/B-27^-ins^ media and spontaneously beating clusters were visible by day 9 onwards. To enrich the HES3-CM population the beating clusters were kept in DMEM (no glucose) media (Gibco) supplemented with 4 mM sodium DL-lactate up to day 12. Generated HES3-CMs maintained either in RPMI/B-27^-ins^ media or in iCell cardiomyocyte maintenance media (Cellular Dynamics, Madison, WI, USA).

### 2.4. RNA Isolation and Quantitative RT-PCR

To analyse the mRNA expression, cells were homogenised with QIAzol lysis reagent (QIAGEN, Hilden, Germany), and the total RNA was extracted using the miRNeasy Mini Kit (QIAGEN, Hilden, Germany) according to the manufacturer’s instructions (for more details see Supplementary Materials). All of the 27 target genes are listed in Figure S3A.

### 2.5. Action Potential Measurements

For action potential (AP) recordings, day 30 HES3-CMs were dissociated into single cells and plated on sterile 0.1% gelatin-coated glass coverslip. 48 h post-plating, APs were recorded by the whole-cell current-clamp technique using PULSE program and EPC 9 amplifier (HEKA) as we described previously [22] (for brief description see Supplementary Materials).

### 2.6. Calcium Imaging

Calcium imaging was performed in HES3-CMs loaded with 5µM Cal-520 AM (AAT Bioquest, Sunnyvale, CA, USA) according to the manufacture protocol with modifications as described previously [23]. Briefly, cells were cultured in p35 plates with 0.08 mm thin glass coverslip bottom and incubated with 1 ml of loading dye solution containing Cal-520 AM at 37 °C for 15 min in dark. Videos and line scans were captured through the Olympus FV1000 Microscope (Olympus, Tokyo, Japan).

### 2.7. Generation of WNT Reporter Human iPSC Line

The IMR90 (hiPS) cells were expanded on Matrigel in iPS-Brew XF medium for a few days. After reaching desired confluency, the cells were transfected with 5 µg of 7TGP plasmid (7TGP was a gift from Roel Nusse (Addgene plasmid # 24305)) consisting 7×Tcf-eGFP cassette using ViaFect^TM^ Transfection Reagent (E498A, Promega, Madison, WI, USA). Positive clones were then selected by adding 1 μg/ml Puromycin for 2 weeks. After the selection, GFP positive clones were picked up for expansion and WNT reporter assay.

### 2.8. Teratoma Analysis

The teratoma assay was performed on SCID (Rag2^−^/^−^common gamma^−^/^−^) mice, as described previously [24]. The animal experiments have been approved by the Universitätsklinikum Köln (Institutional Ethics Review Board reference number 01-090) and the governmental animal care and use office (Landesamt für Natur, Umwelt und Verbraucherschutz Nordrhein-Westfalen, Recklinghausen, Germany (reference number 84-02.04.2012.A417 LANUV). For more experimental details see Supplementary Materials.

### 2.9. Short Interfering RNA-Targeted Gene Silencing

Pool of COX-1 (M-004556-00-0005) and COX-2 (M-004557-01-0005) siRNAs (Dharmacon, Freiburg, Germany) were used to interfere with human COX-1 and COX-2 expression in IMR90 cells. An equivalent amount of scrambled siRNA was used as a negative control. The IMR90 cells were seeded into 60-mm culture dishes and treated with CHIR99021 from day 0 to day 1. On day 2 cells were transfected with COX-1 and COX-2 siRNA and scrambled siRNA using Magnetofectamine™ (OZBiosciences, Marseille, France) according to the manufacturer’s protocol.

### 2.10. Statistical Analysis

All the experiments were performed in triplicates and data are presented as mean ± SEM or ± SD. Unpaired two-tailed Student’s *t* test was used to calculate statistical significance and *p* values ≤ 0.05 were considered as statistically significant.

## 3. Results

### 3.1. Sulindac Efficiently Promotes Cardiogenesis from hESCs

**Figure 1:**
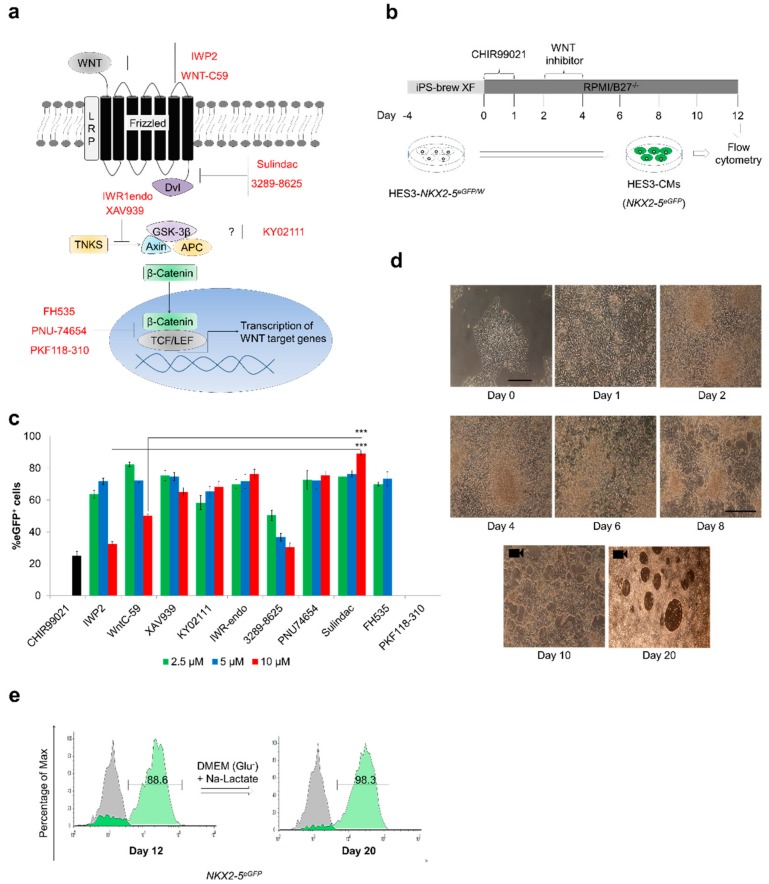
Sulindac efficiently promotes cardiogenesis in human embryonic stem cells (hESCs): (**a**) Schematic representation of the Wnt signaling transduction pathway. When activation occurs via binding of Wnt ligand to Frizzled receptor, beta catenin translocates into the nucleus and activates transcription of Wnt target genes. Different small molecule Wnt inhibitors interact with different target proteins in Wnt pathway leading to phosphorylation and degradation of beta catenin and inhibition of transcription of Wnt target genes; (**b**) Protocol for in vitro cardiac differentiation using Wnt signaling modulators. Undifferentiated hPSC colonies were maintained in iPS-brew XF media from days −4 to 0. Mesendodermal differentiation was initiated in the first phase by changing the culture media to RPMI-B27^-/-^ media containing CHIR99021 (10 μM). In the second phase, cells were cultured in the presence of Wnt inhibitors (2.5 μM, 5 μM or 10 μM) from day 2 to 4. Spontaneously beating cardiac clusters were observed from day 9 onwards; (**c**) Proportion of eGFP^+^ cells observed with different Wnt inhibitors at different concentrations from 3 independent experiments. Cardiac differentiation was carried out according to (**b**) and the proportion of eGFP^+^ cells was measured on day 12 by flow cytometry (Error bars, ±SEM; *n* = 3 independent biological replicates, Student’s *t* test, * *p* ≤ 0.05, ** *p* ≤ 0.01, *** *p* ≤ 0.001); (**d**) Representative phase-contrast images of HES3 cells taken over the 20 day period of Sulindac-induced differentiation show pluripotent HES3 cells and cell in cardiac transition. Scale bar, 50 µm. Videos of day 10 and day 20 CMs are provided in Video-1 and Video-2 respectively; (**e**) Representative FACS analyses for percent eGFP^+^ cells before lactate treatment (day 12) and after lactate treatment (day 20). Control (Gray) indicates undifferentiated HES3 cells.

Cardiac differentiation under monolayer differentiation conditions [1] has been monitored using HES3 reporter line [19]. Ten small molecule WNT inhibitors (IWP2, Wnt-C59, XAV939, IWR1endo, KY02111, Sulindac, FH535, 3289-8625, PKF118-310 and PNU-74654) were assessed at three test concentrations (2.5 µM, 5 µM and 10 µM) for their potential to generate functional CMs (HES3-CMs) (Figure 1).

Despite their differences in inhibition mechanisms (Figure 1a) these small molecules efficiently inhibit Wnt signaling and transcription of Wnt target genes. For example, IWP2 and Wnt-C59 inhibit PORCN involved in Wnt synthesis and Sulindac and 3289-8625 inhibit Wnt signaling through blockade of the Dishevelled (Dvl-PDZ domain) whereas XAV939 and FH535 block Wnt signaling by interacting with TNKS and TCF/LEF, respectively. Cardiac differentiation was induced using the protocol outlined in Figure 1b. In brief, the undifferentiated HES3 cells were treated with CHIR99021 (day 0–1) followed by Wnt inhibitor (day 2–4) and maintained in drug-free media from day 4 onwards with media change on every alternate day. At day 12, eGFP^+^ cells were quantified using flow cytometry. Nine out of 10 tested Wnt inhibitors induced cardiogenesis in HES3 cells with eGFP^+^ cell populations ranging from 60–95% and Sulindac with 88–92% eGFP^+^ cells was found to be the most effective small molecule in our model (Figure 1c). Phase-contrast images taken over the 20 day period of Sulindac induced differentiation show pluripotent HES3 cells and cell in cardiac transition (Figure 1d), spontaneously beating clusters of Sulindac-derived HES3-CMs were observed from day 8 onwards (Video-1). To further enrich this CMs population, the beating cells were cultured in glucose-free DMEM supplemented with sodium lactate from day 12–20, sequential flow cytometry analysis confirmed the enrichment in CMs with 98–99% eGFP^+^ cells (Figure 1e) (Video-2). These CMs can be maintained in synchronously beating cardiac sheets for >120 days (Video-3). To further confirm the purity of the CM cultures and terminal differentiation in CMs, we injected undifferentiated HES3 cells and Sulindac-derived HES3-CMs into immunosuppressed mice. As shown in Figure S1A, we observed tumour formation in mice injected with undifferentiated HES3 cells whereas mice injected with purified Sulindac-derived HES3-CMs did not develop tumours. Lack of efficacy across multiple hESC and hiPSC lines is the major drawback associated with several established protocols [1]. To explore the applicability of Sulindac-based cardiac differentiation protocol, we tested our protocol with three more hPSC lines (IMR90: hiPSC line; ES4SKIN: a patient-derived hiPSC line, and H9: hESC line) at passages between 10 and 25. We observed that Sulindac treatment efficiently induced cardiac differentiation with 50–70% cardiac troponin T positive (cTnT^+^) cells (Figure S1B). Taken together data confirm that Sulindac promotes cardiomyogenesis in hESCs and hiPSCs.

### 3.2. Sulindac Treatment was Sufficient to Accelerate Cardiac Differentiation from hESCs and Maturation of Cardiomyocytes

**Figure 2:**
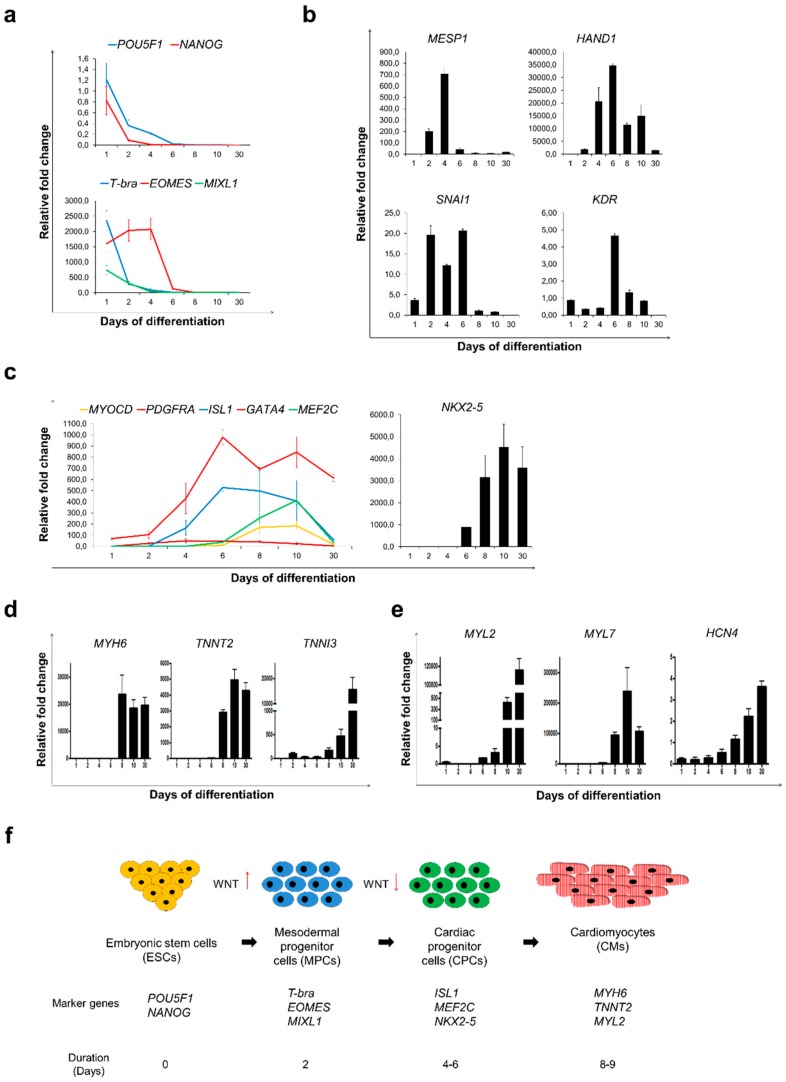
Sulindac treatment was sufficient to accelerate cardiac differentiation and maturation of cardiomyocytes. Gene expression analysis by qRT-PCR was performed during Sulindac-based cardiac differentiation protocol for; (**a**) markers for pluripotency: *POU5F1* and *NANOG* (Top) and early mesoderm: *T-bra*, *EOMES* and *MIXL1* (bottom), (**b**) markers for cardiac mesoderm: *MESP1*, *HAND1*, *SNAI1* and *KDR*, (**c**) markers for cardiac progenitor cells: *MYOCD*, *PDGFRA*, *ISL1*, *GATA4*, *MEF2C* and *NKX2-5*, (**d**) Cardiac-specific genes *MYH6, TNNT2* and *TNNI3*, (**e**) ventricular, atrial and nodal sub-type-specific markers *MYL2, MYL7* and *HCN4*. Samples collected for this analysis were photographed and presented in Figure 1d. All Ct values are normalised using GAPDH and relative fold change was calculated using Day 0 HES3 cells as control. Error bars, ±SEM; *n* = 3 independent biological replicates; (**f**) Schematic representation of transition of HES3 cells from pluripotent stem cells to cardiomyocytes represented by stage-specific expressed marker genes.

Using custom designed RT^2^ profiler PCR arrays, we performed step-by-step molecular analysis of Sulindac-induced cardiac differentiation to unravel the rapid changes in gene expressions leading to cardiogenesis. Figure S2A shows the heat map representing the expression profile changes in the 27 reference pluripotency, mesoderm, and cardiac development marker genes during the differentiation protocol. We observed significant downregulation of pluripotency markers *POU5F1* and *NANOG* with simultaneous upregulation of early mesodermal and primitive streak markers like *T-bra*, *EOMES* and *MIXL1* from day 1–4, transforming hESCs into mesodermal progenitor cells (MPCs) [25,26,27,28,29] (Figure 2a).

Marked upregulation of mesoderm-specific transcription factors and markers of cardiac progenitors such as *MESP1*, *HAND1*, *SNAI1* and *KDR* from days 4-6 confirmed hESCs transition into cardiac progenitor cells (CPCs) [30,31,32,33] (Figure 2b). Additionally, we also detected noticeable upregulation in key CPC markers, like *MYOCD*, *PDGFR-α*, *GATA4*, *MEF2C* and *NKX2.5* [34] from day 6–10. Up-regulation of *ISL1*, a marker for induction of secondary heart field (SHF) [35] was also detected from day 4 onwards which gradually decreased by day 30 (Figure 2c). These sequential alterations in the mRNA levels confirmed the cardiac differentiation, yielding pure CM population (>90% eGFP^+^ cells) by day 12.

Noticeable, upregulation in cardiac markers like, Myosin heavy chain (*MYH6*), Troponin T2 (*TNNT2*) and Troponin I3 (*TNNI3*) was observed from day 6 onwards (Figure 2d). The mRNA expression pattern of two major isoforms of myosin light-chain 2, *MYL2* (*MLC2v*) and *MYL7* (*MLC2a*) which mark ventricular- and atrial-like cells respectively [36,37] and *HCN4* which marks pacemaker-/nodal-like cells [38], confirmed the presence of three subtypes of CMs (Figure 2e). Consistent with a previous report [8], we observed that the maximum numbers of the eGFP^+^ cells were primarily expressing *MYL7* by day 10, representing atrial-like and immature ventricular CMs. However, by day 30, the expression of *MYL7* decreased with a simultaneous rapid increase in *MYL2*, suggesting a possible increase in ventricular-like cells and maturation of CMs [39]. In addition, the upregulation of *SIRPA* and *VCAM1* mRNA levels from day 10 onwards suggest that Sulindac treatment leads to transformation of CPCs (NKX2-5^+^) to myocardial committed cells (NKX2-5^+^/SIRPA^+^) and then to functional CMs (NKX2-5^+^/SIRPA^+^/VCAM1^+^) [40] (Figure S2B). Taken together, we identified four intermediate stages of cardiac differentiation based on the mRNA expression profiles of key marker genes: (1) ESCs expressing pluripotency markers like *POU5F1* and *NANOG*, (2) MPCs expressing *T-bra*, *EOMES,* and *MIXL1*, (3) CPCs expressing *NKX2-5*, *ISL1* and *GATA4* and (4) functional CMs expressing *MYH6*, *TNNT2* and *TNNI3* (Figure 2f).

### 3.3. Structural and Functional Characterisation of Sulindac-Derived HES3-CMs

**Figure 3:**
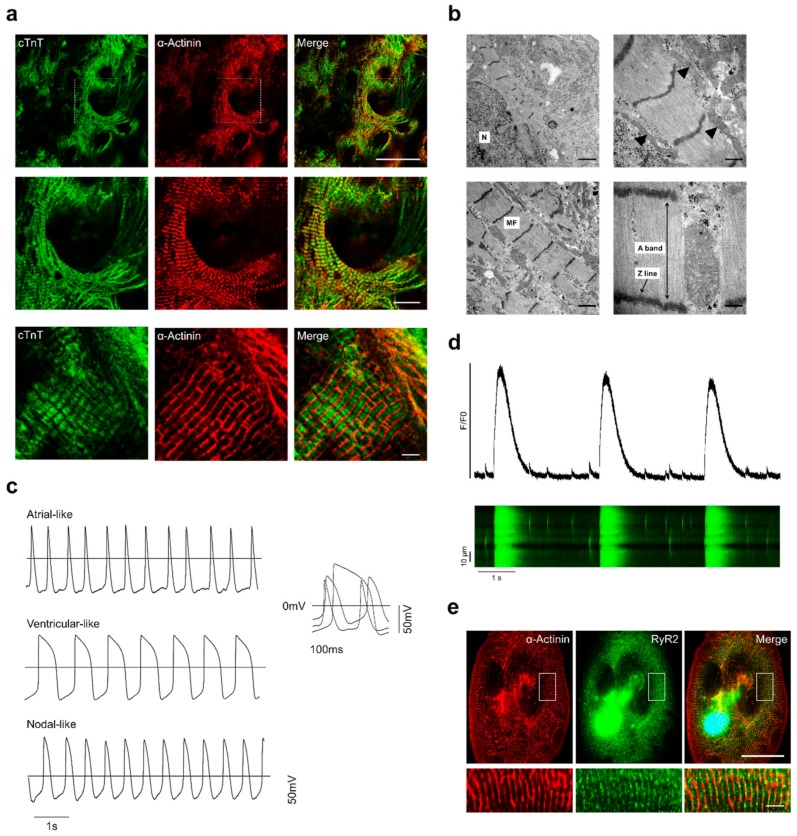
Structural and functional characterisation of Sulindac-derived HES3-CMs: (**a**) Immunofluorescence staining of 30 day HES3-CMs with antibodies to the indicated proteins. Scale bars, 50 μm (Top panel), 20 μm (middle panel) and 5 μm (bottom panel); (**b**) TEM images of Sulindac-derived HES3-CMs. Upper left and right images, N = Nucleus, mitochondria (black arrowheads). Bottom left and right images, MF = Myofibrils, detailed assembly of myofibrillar bundles showing Z line and A band. Scale bar = 1000 nm (Left Top and bottom), 500 nm (Right Top and bottom); (**c**) Representative traces of spontaneous atrial-, ventricular- and nodal-like action potential (AP) in patch-clamp recordings from day 30 CMs; (**d**) Typical Ca^2+^ transients recorded from 30 day HES3-CMs using Cal-520AM calcium indicator. Fluorescence profiles (top) is taken from individual line scan image (bottom). Also, see Video 3; (**e**) Immunofluorescence staining of 30 day HES3-CMs with antibodies to the indicated proteins. Scale bars, 50 μm (Top panel) and 5 μm (bottom panel).

To determine the success of our Sulindac-derived cardiac differentiation protocol, we performed detailed characterisation of the resulting CMs. Immunofluorescence staining of day 30 HES3-CMs with anti-sarcomeric α-Actinin and anti-cTnT antibodies demonstrated a typical striation pattern and organised myofilaments similar to those of adult heart tissue (Figure 3a).

Transmission electron microscopy also revealed structural features of Sulindac-derived HES3-CMs such as bundles of myofilaments and sarcomere with Z-line, A-band and abundant mitochondria (Figure 3b). To assess the electrophysiological (EP) properties we performed single cell patch-clamp recordings (Table S1). Based on AP properties like action potential amplitude (APA), action potential duration (APD) at 10%, 50%, or 90% of repolarisation and dV/dtmax, three subtypes of CMs were confirmed (Figure 3c) [41]. Final data analysis revealed CM population comprised of 60–70% ventricular-like, 20–25% atrial-like and 10–15% nodal-like cells (Table S2). We also investigated intracellular Ca^2+^ dynamics in Sulindac-derived HES3-CMs by recording Ca^2+^ transients using Cal-520 AM calcium indicator. Line-scan images and data analysis showed Ca^2+^ oscillations and Ca^2+^ transient parameters comparable to that of native CMs (Figure 3d) (Table S3). Furthermore, we also observed spontaneous localised intracellular Ca^2+^ release events “calcium sparks” (Figure S2C) (Video-4) released through ryanodine receptor 2 (*RYR2*) that controls calcium-induced calcium release (CICR) in CMs [42]. RT-PCR and Immunofluorescence staining of ryanodine receptor 2 (*RYR2*) confirmed gradual increase in *RYR2* mRNA levels (Figure S2D) and the presence of functional RyR2 channels in Sulindac-derived HES3-CMs (Figure 3e). Collectively, these results show that Sulindac-derived HES3-CMs are structurally and electrically mature and functional.

### 3.4. Sulindac-Derived HES3-CMs Offer a Human-Relevant in vitro Cardiotoxicity Screening Model

**Figure 4:**
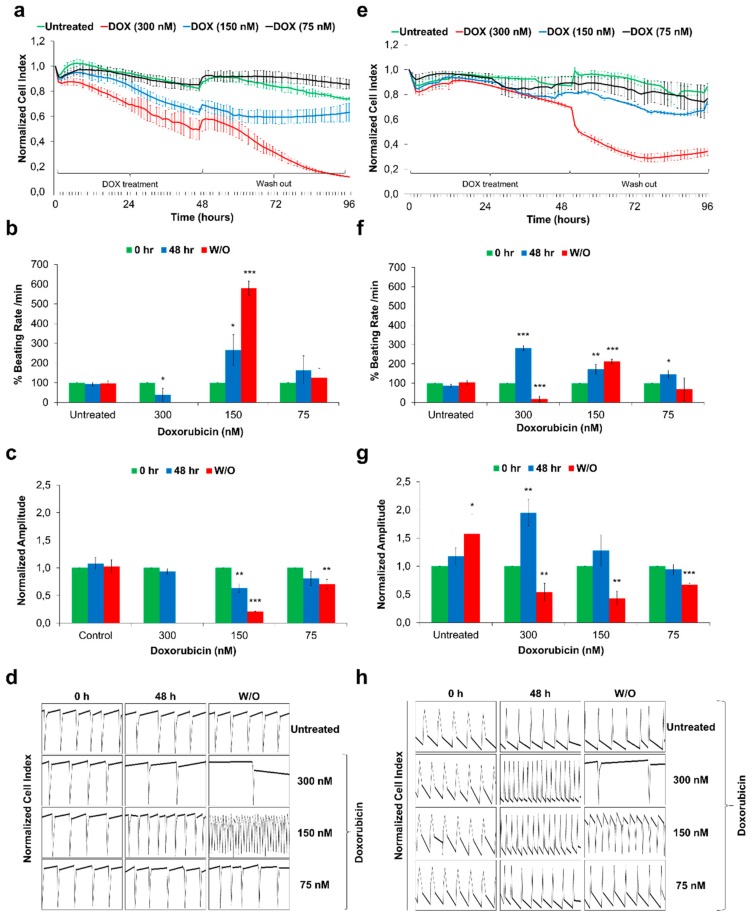
Functional studies of Doxorubicin-exposed Sulindac-derived HES3-CMs and hiPSC-CMs using the xCELLigence RTCA Cardio system. The HES3-CMs and hiPSC-CMs were cultured in fibronectin-coated E-cardio plates and treated with Doxorubicin for 48 h followed by 48 h drug washout (W/O). Cells were monitored using xCELLIgence RTCA Cardio system for real time changes; (**a**,**e**) The representative graphs displays DOXO exposure induces cytotoxicity and drop in Cell Index values in HES3-CMs and hiPSC-CMs respectively. Error bars, ±SEM; *n* = 3 independent biological replicates; (**b**,**f**) The representative graphs displays DOXO exposure induces changes in % beating rates in HES3-CMs and hiPSC-CMs respectively. Error bars, ±SEM; *n* = 3 independent biological replicates; (Student’s *t* test, * *p* ≤ 0.05, ** *p* ≤ 0.01, *** *p* ≤ 0.001) (**c**,**g**) The representative graphs displays DOXO exposure induces changes in beating amplitude in HES3-CMs and hiPSC-CMs respectively. Error bars, ±SEM; *n* = 3 independent biological replicates; (Student’s *t* test, * *p* ≤ 0.05, ** *p* ≤ 0.01, *** *p* ≤ 0.001) (**d**,**h**) Representative 12 s beating traces of HES3-CMs and hiPSC-CMs after DOXO exposures and during drug washout. Y-axis represents Normalised Cell Index. Beating activity demonstrates the development of arrhythmic beating upon DOXO exposure. Error bars, ±SEM; *n* = 3 independent biological replicates.

Functional CMs derived from hPSCs offer a human-relevant in vitro cardiotoxicity screening platform. In this context, the cardiotoxic effects of Doxorubicin (DOXO) were assessed using recently reported xCELLigence Real-Time Cell Analyser (RTCA) based screening platform [43]. DOXO is a anthracycline class anticancer drug, well-known to induce acute cardiotoxicity leading to heart failure [44]. We treated commercially available hiPSC-CMs (iCell Cardiomyocytes) and Sulindac-derived HES3-CMs (day 30) with a single dose of 300 nM, 150 nM and 75 nM DOXO for 48 h followed by 48 h of drug washout. Raw data for cell viability, beating rate, beating amplitude and beating profile were acquired using the xCELLigence RTCA Cardio system and analysed using RTCA Cardio software version 1.0. Figure 4 represents data obtained from Sulindac-derived HES3-CMs (Figure 4a–d) and hiPSC-CMs (Figure 4e–h).

In brief, we observed significant drop in cell index when CMs were treated with 300 nM DOXO (Figure 4a,e) whereas CMs treated with 150 nM DOXO showed a striking irreversible increase in beating rate with a significant decrease in beating amplitude (Figure 4b,f, and c,g). Further analysis of beating profiles of CMs from control and DOXO treated group confirmed the arrhythmic beating in DOXO treated CMs (Figure 4d,h). Our data clearly shows that Sulindac-derived HES3-CMs are more sensitive towards known cardiotoxicants like DOXO compared to hiPSC-CMs. Though factors like differentiation protocol used, culture conditions, batch-to-batch variations, maturity and age of CMs could explain these differences, our findings demand further in-depth analysis of hESC-CMs and hiPSC-CMs based screening models.

### 3.5. Cyclooxygenase Inhibition Plays an Important Role in Cardiomyocytes Differentiation

**Figure 5:**
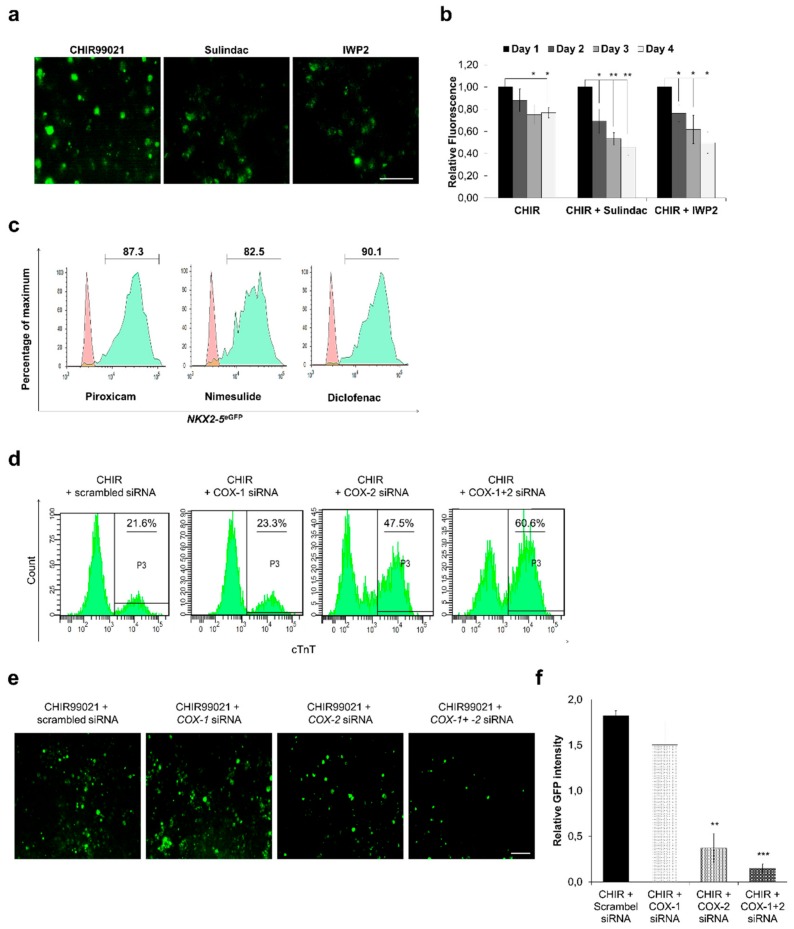
Cyclooxygenase inhibition plays an important role in cardiomyocytes differentiation. TCF reporter assay using IMR90-WNT reporter line: (**a**,**b**) The effect of Sulindac and IWP2 was examined. On day 0, CHIR99021 (10 μM) was added to activate Wnt signaling and TCF promoter activity from day 2 to day 4 cells were treated with Sulindac and IWP2. Fluorescence was recorded and images were captured on day 4. Scale bar, 100 μm (**a**), Error bars, ±SD; *n* = 3 independent biological replicates, (Student’s *t* test, * *p* ≤ 0.05, ** *p* ≤ 0.01, *** *p* ≤ 0.001) (**b**); (**c**) Representative FACS analyses for percent eGFP^+^ cells generated using Nimesulide, Diclofenac and Piroxicam. Control (red) indicates undifferentiated HES3 cells. (**d**) Representative FACS analyses for percent cTnT^+^ cells generated using COX-1, COX-2 and COX-1+2 siRNA. Undifferentiated IMR90 cells were used as control. (**e**,**f**) IMR90-WNT reporter cells were transfected with COX-1, COX-2 and COX-1+2 siRNAs after treatment with CHIR99021. Fluorescence images were captured on day 4 and analysed using ImageJ to obtain relative GFP intensities. Scale bar = 50 μm, Error bars, ±SEM; *n* = 3 independent biological replicates, (Student’s *t* test, * *p* ≤ 0.05, ** *p* ≤ 0.01, *** *p* ≤ 0.001).

Sulindac has been reported to suppress Wnt3A-induced β-catenin signaling by binding to PDZ domain of Dvl [45,46]. To find out whether Sulindac inhibits Wnt signaling in hPSCs we generated IMR90-WNT reporter cell line which possesses a 7×Tcf-eGFP reporter cassette [47]. The confocal imaging and fluorescence measurements confirmed downregulation of Wnt signaling after Sulindac treatment, IWP2 was used as a positive control (Figure 5a,b).

Our initial FACS data also showed that, in comparison to other tested Wnt inhibitors including 3289-8625, which also binds and inhibits Dvl-PDZ domain, Sulindac yields a significantly higher number of eGFP^+^ cells (Figure 1a,c). These data suggest that, Sulindac promotes cardiogenesis not only by inhibiting Wnt signaling but also by a yet unexplored secondary mechanism. Sulindac is a NSAID and non-selectively inhibits both isoforms of cyclooxygenase (COX) namely, cyclooxygenase-1 (COX-1) and cyclooxygenase-2 (COX-2). We hypothesised that this inhibition of COX by Sulindac could also play a role in cardiogenesis. To validate our hypothesis, we knocked down the COX-1 and COX-2 expression in hPSCs either by using small molecule COX-inhibitors like Piroxicam, Nimesulide and Diclofenac or by transfecting pool of siRNAs directed against COX-1 and COX-2. Quantification of eGFP^+^ cells on day 12 confirmed the cardiac differentiation in COX-inhibitor treated HES3 cells with 85–90% eGFP^+^ cells (Figure 5c) similar results were observed in IMR90 cells (data not shown). We next transfected IMR90 and HES3 cells with COX-1 and COX-2 directed siRNA pools and measured the cTnT^+^ and eGFP^+^ cells on day 12 using FACS. Our data show that inhibition of COX-1 alone does not improve cardiac differentiation significantly however inhibition of COX-2 alone or inhibition of COX-1 and COX-2 together significantly improves cardiac differentiation and increase the yield of CMs (Figure 5d) (Figure S3A). These findings suggest that, controlled and stage-specific inhibition of COX promotes cardiomyogenesis from hPSCs in vitro.

To further explore the molecular mechanism involved in cardiac differentiation and COX inhibition, we transfected IMR90-WNT reporter line with COX-1 and COX-2 siRNAs. The fluorescence images were captured and analysed using ImageJ to obtain relative GFP intensity values. Our data show that inhibition of COX-1 had no significant effect on WNT pathway activity whereas inhibition of COX-2 alone or COX-1 and COX-2 together showed significant downregulation in Wnt pathway activity when compared with scramble siRNA control (Figure 5e,f). This finding explains higher yields of CMs in Sulindac and Diclofenac as they inhibit both COX isoforms. These data also indicate that COX pathway acts upstream to Wnt pathway and both pathways collectively contribute in cardiac differentiation.

## 4. Discussion

Landmark discovery to reprogram somatic cells to generate iPSCs have opened up new avenues for developing more physiologically relevant platforms for drug discovery and patient-specific cell therapies [48]. Several recent studies show that hiPSC-CMs can be used in regenerative medicine [49] and an in vitro cardiotoxicity screening thereby offering human-relevant alternative for animal testing [43,50,51]. However, high costs associated with commercially available hiPSC-CMs limit their use for most academic research. In addition, line-to-line and batch-to-batch variations result in alterations in expression of key ion channels in hiPSC-CMs hence demands careful evaluation of the data [6,7,52,53]. Differences in the sensitivity towards several drugs amongst hESC-CMs and hiPSC-CMs also suggest that the screening-assays using hiPSC-CMs must be applied with caution [2,7].

Here, we developed an optimised cardiac differentiation protocol by screening 10 small molecule Wnt inhibitors using HES3 cells [19]. Quantification of *NKX2-5* driven eGFP expression on day 12 confirmed cardiac differentiation in nine tested Wnt inhibitors with 30–95% eGFP^+^ cells and spontaneously beating clusters were visible by day 9 onwards. Sulindac induced ~92% eGFP^+^ cells and is thereby identified as a potent cardiogenic agent. We further demonstrated that Sulindac-induces cardiac differentiation in another hESC cell line (H9) and 2 different hiPSCs (IMP90 and ES4SKIN) and confirmed the cardiac differentiation with 50–70% cTnT^+^ cells by day 12. Although we observed some variations in the efficiency of cardiac differentiation between hiPSC and hESCs, the pattern of differentiation was similar among all three tested lines.

Consistent with previous reports, we observed that CHIR99021 treatment at an early phase, day 0–1, downregulated pluripotency genes *POU5F1*, *NANOG* and generate primitive streak cells expressing marker genes *T-bra*, *MIXL1* and *EOMES* [1,8]. Concurrent upregulation of *MESP1* and *PDGFR-α^+^/KDR^+^* cells confirmed the induction of cardiac mesoderm and generation of CPCs after Sulindac treatment [54]. In addition, RT-PCR data also confirmed upregulation of CPC markers like *ISL1*, *GATA4*, *NKX2-5*, *MEF2C*, *MYOCD*, *HAND1,* and *SNAI1* from day 4–8. Combined, these genetic changes then activate cardiac structural genes such as *TNNT2*, *TNNI3,* and *MYH6* leading to the generation of beating CMs. These data also confirm that Sulindac efficiently promotes cardiac differentiation by inducing genes involved in a highly conserved network that controls initial mesodermal differentiation, proliferation of MPCs and CPCs and maturation of CMs [55]. Using Sulindac we efficiently generated CMs within 20 days in a defined medium supplemented with B27 without insulin which can be maintained in cultures for up to 120 days as synchronously beating cardiac sheets. Moreover, our protocol proved beneficial over other known cardiac differentiation methods as it requires no genetic modification and it could be applicable to many existing hPSC lines.

The hPSC-CM population at an early phase of differentiation is largely composed of *MYL7*^+^/*MYL2*^-^, immature and human fetal-like CMs which then mature and transform into *MYL7*^-^/*MYL2*^+^ CMs [1,8,56]. Based on the expression profiles of *MYL2* and *MYL7* mRNAs, we observed similar transition of HES3-CMs in our study. Electrophysiological characterisation confirmed that Sulindac-derived CMs exhibited AP characteristics similar to that of mature CMs with ventricular-like CMs being predominant, making >65% of total cell population by day 30 and >75% by day 60 (data not shown). These findings suggest that Sulindac-derived HES3-CMs exhibit physiologically relevant EP properties.

In CMs, Ca^2+^ transients act as a chemical signal that transduces electrical depolarisation to mechanical movement, also known as excitation-contraction (EC) coupling. Cardiac EC coupling is governed by a Ca^2+^-induced Ca^2+^ release (CICR) mechanism and short-lived Ca^2+^ signals (sparks) were revealed as elementary events during EC coupling [57,58]. Ca^2+^ transient recordings performed with Sulindac-derived HES3-CMs revealed Ca^2+^ oscillations comparable to that of mature CMs and local, short-lived Ca^2+^ sparks suggesting the presence of functional SR-Ca^2+^ stores. Additionally, upregulation of *RYR2* mRNA levels and immunostaining indicated the presence of functional ryanodine receptors which in combination with SR-Ca^2+^ stores contribute to Ca^2+^ transients [59,60] and demonstrates mature and functional CMs.

The hPSC-CMs generated using most known protocols consist of contaminating population of undifferentiated or non-cardiac cells which would increase the tumour formation after transplantation hence limit their use in clinical applications [61,62]. Culturing hPSC-CMs in glucose-depleted culture media containing abundant lactate shown to enrich the CMs to up to 99% [63]. Using a similar approach, we enriched Sulindac-derived HES3-CMs to >97% purity, the purified CMs did not form tumours after transplantation into immunosuppressed Rag^-/-^ mice confirming pure terminally differentiated population of CMs.

Besides clinical use, hPSC-CMs are an important tool for an in vitro cardiotoxicity studies. Recent studies from our and several other groups have shown that commercially available hiPSC-CMs can be used for an in vitro toxicity studies and offer a human-relevant alternative for animal models [43,64,65,66]. Here we show that Sulindac-derived HES3-CMs in combination with xCELLigence Real-Time Cell Analyser (RTCA) offer a robust, sensitive and reliable model for an in vitro cardiotoxicity assessment. We also show that Sulindac-derived HES3-CMs respond to known cardiotoxicants like Doxorubicin, demonstrating functionally mature and electrophysiologically active CMs.

The non-steroidal anti-inflammatory drug (NSAID)-Sulindac, inhibits cyclooxygenase-1 (COX-1) and cyclooxygenase-2 (COX-2) and is well studied for its anti-inflammatory and antineoplastic potential [20,21]. To ascertain the role of COX-1 and COX-2 in cardiogenesis, we knocked down COX-1 and COX-2 levels in HES3 and IMR90 cells by treatment with different NSAIDs (e.g., Piroxicam, Nimesulide and Diclofenac) and by using pool of siRNAs directed towards COX-1 and COX-2. Quantification of eGFP^+^ cells on day 12 confirmed the cardiogenesis with 85–90% eGFP^+^ cells showing for the first time that NSAIDs holds cardiomyogenic potential and can differentiate hPSCs into functional CMs, in vitro. Our findings coincide with recent study from Muraoka et.al. in which diclofenac was shown to enhance cardiac reprogramming via suppression of COX-2 [67]. Moreover, our siRNA study shows that inhibition of COX-2 alone or COX-1 and COX-2 together can efficiently induce cardiac differentiation whereas inhibition of COX-1 alone did not show significant improvement in cardiac differentiation. Several studies have shown that NSAIDs can inhibit Wnt signalling in cancer cells by targeting c-Met and 5-lipoxygenase [68]. However, no conclusive data on the interactions of Wnt and COX signalling in stem cells is available. Our study for the first time shows that siRNA directed inhibition of COX-2 alone or COX-1 and COX-2 together significantly downregulate Wnt pathway activity in hPSCs. These findings also suggest that in stem cells COX pathway might be an upstream event into Wnt signalling pathway.

## Supplementary Materials

The following are available online at https://www.mdpi.com/2073-4409/9/3/554/s1, Figure S1: Tumorigenicity of Sulindac-derived HES3-CMs and applicability of Sulindac-based cardiac differentiation protocol; Figure S2: Differentiation and characterisation of Sulindac-derived HES3-CMs; Figure S3: Differentiation of HES3 cells using COX siRNA. Table S1 and S2: Electrophysiological characterisation of Sulindac-derived HES3-CMs. Table S3: Calcium transient properties of Sulindac-derived HES3-CMs. Video 1 and 2: HES3 cells were differentiated in RPMI+B27-/- medium using Sulindac-based cardiac differentiation protocol (Figure 1b). Videos 1 and 2 show CMs on day 10 and day 20 respectively; Video 3: Sulindac-derived HES3-CMs maintained for >60 days are shown as synchronously beating cardiac sheets; Video 4: Sulindac-derived HES3-CMs were loaded with calcium indicator Cal-520 AM (5 µM) at room temperature for 15 min in dark. The video was recorded on day 36.

## References

[1] Burridge P.W., Matsa E., Shukla P., Lin Z.C., Churko J.M., Ebert A.D., Lan F., Diecke S., Huber B., Mordwinkin N.M. (2014). Chemically defined generation of human cardiomyocytes. Nat. Meth..

[2] Foldes G., Matsa E., Kriston-Vizi J., Leja T., Amisten S., Kolker L., Kodagoda T., Dolatshad N.F., Mioulane M., Vauchez K. (2014). Aberrant alpha-adrenergic hypertrophic response in cardiomyocytes from human induced pluripotent cells. Stem Cell Rep..

[3] Gore A., Li Z., Fung H.L., Young J.E., Agarwal S., Antosiewicz-Bourget J., Canto I., Giorgetti A., Israel M.A., Kiskinis E. (2011). Somatic coding mutations in human induced pluripotent stem cells. Nature.

[4] Lundy S.D., Zhu W.Z., Regnier M., Laflamme M.A. (2013). Structural and functional maturation of cardiomyocytes derived from human pluripotent stem cells. Stem Cells Dev..

[5] Sepac A., Si-Tayeb K., Sedlic F., Barrett S., Canfield S., Duncan S.A., Bosnjak Z.J., Lough J.W. (2012). Comparison of cardiomyogenic potential among human ESC and iPSC lines. Cell Transplant..

[6] Goversen B., van der Heyden M.A.G., van Veen T.A.B., de Boer T.P. (2018). The immature electrophysiological phenotype of iPSC-CMs still hampers in vitro drug screening: Special focus on IK1. Pharm. Ther..

[7] Sheng X., Reppel M., Nguemo F., Mohammad F.I., Kuzmenkin A., Hescheler J., Pfannkuche K. (2012). Human pluripotent stem cell-derived cardiomyocytes: Response to TTX and lidocain reveals strong cell to cell variability. PLoS ONE.

[8] Lian X., Hsiao C., Wilson G., Zhu K., Hazeltine L.B., Azarin S.M., Raval K.K., Zhang J., Kamp T.J., Palecek S.P. (2012). Robust cardiomyocyte differentiation from human pluripotent stem cells via temporal modulation of canonical Wnt signaling. Proc. Natl. Acad Sci. USA.

[9] Tohyama S., Fujita J., Fujita C., Yamaguchi M., Kanaami S., Ohno R., Sakamoto K., Kodama M., Kurokawa J., Kanazawa H. (2017). Efficient Large-Scale 2D Culture System for Human Induced Pluripotent Stem Cells and Differentiated Cardiomyocytes. Stem Cell Rep..

[10] Sachinidis A., Schwengberg S., Hippler-Altenburg R., Mariappan D., Kamisetti N., Seelig B., Berkessel A., Hescheler J. (2006). Identification of small signalling molecules promoting cardiac-specific differentiation of mouse embryonic stem cells. Cell Physiol. Biochem..

[11] Happe C.L., Engler A.J. (2016). Mechanical Forces Reshape Differentiation Cues That Guide Cardiomyogenesis. Circ. Res..

[12] Meganathan K., Sotiriadou I., Natarajan K., Hescheler J., Sachinidis A. (2015). Signaling molecules, transcription growth factors and other regulators revealed from in-vivo and in-vitro models for the regulation of cardiac development. Int. J. Cardiol..

[13] Willems E., Spiering S., Davidovics H., Lanier M., Xia Z., Dawson M., Cashman J., Mercola M. (2011). Small-molecule inhibitors of the Wnt pathway potently promote cardiomyocytes from human embryonic stem cell-derived mesoderm. Circ. Res..

[14] Minami I., Yamada K., Otsuji T.G., Yamamoto T., Shen Y., Otsuka S., Kadota S., Morone N., Barve M., Asai Y. (2012). A Small Molecule that Promotes Cardiac Differentiation of Human Pluripotent Stem Cells under Defined, Cytokine- and Xeno-free Conditions. Cell Rep..

[15] Dubois N.C., Craft A.M., Sharma P., Elliott D.A., Stanley E.G., Elefanty A.G., Gramolini A., Keller G. (2011). SIRPA is a specific cell-surface marker for isolating cardiomyocytes derived from human pluripotent stem cells. Nat. Biotechnol..

[16] Hentze H., Soong P.L., Wang S.T., Phillips B.W., Putti T.C., Dunn N.R. (2009). Teratoma formation by human embryonic stem cells: Evaluation of essential parameters for future safety studies. Stem Cell Res..

[17] Schwach V., Passier R. (2016). Generation and purification of human stem cell-derived cardiomyocytes. Differentiation.

[18] Ban K., Bae S., Yoon Y.-S. (2017). Current Strategies and Challenges for Purification of Cardiomyocytes Derived from Human Pluripotent Stem Cells. Theranostics.

[19] Elliott D.A., Braam S.R., Koutsis K., Ng E.S., Jenny R., Lagerqvist E.L., Biben C., Hatzistavrou T., Hirst C.E., Yu Q.C. (2011). NKX2-5(eGFP/w) hESCs for isolation of human cardiac progenitors and cardiomyocytes. Nat. Methods.

[20] Scheper M.A., Nikitakis N.G., Chaisuparat R., Montaner S., Sauk J.J. (2007). Sulindac induces apoptosis and inhibits tumor growth in vivo in head and neck squamous cell carcinoma. Neoplasia.

[21] Taylor M.T., Lawson K.R., Ignatenko N.A., Marek S.E., Stringer D.E., Skovan B.A., Gerner E.W. (2000). Sulindac Sulfone Inhibits K-ras-dependent Cyclooxygenase-2 Expression in Human Colon Cancer Cells. Cancer Res..

[22] Nembo E.N., Atsamo A.D., Nguelefack T.B., Kamanyi A., Hescheler J., Nguemo F. (2015). In vitro chronotropic effects of Erythrina senegalensis DC (Fabaceae) aqueous extract on mouse heart slice and pluripotent stem cell-derived cardiomyocytes. J. Ethnopharmacol..

[23] Walter A., Saric T., Hescheler J., Papadopoulos S. (2016). Calcium Imaging in Pluripotent Stem Cell-Derived Cardiac Myocytes. Methods Mol. Biol..

[24] Faitschuk E., Hombach A.A., Frenzel L.P., Wendtner C.M., Abken H. (2016). Chimeric antigen receptor T cells targeting Fc mu receptor selectively eliminate CLL cells while sparing healthy B cells. Blood.

[25] Davis R.P., Ng E.S., Costa M., Mossman A.K., Sourris K., Elefanty A.G., Stanley E.G. (2008). Targeting a GFP reporter gene to the MIXL1 locus of human embryonic stem cells identifies human primitive streak-like cells and enables isolation of primitive hematopoietic precursors. Blood.

[26] Den Hartogh S.C., Wolstencroft K., Mummery C.L., Passier R. (2016). A comprehensive gene expression analysis at sequential stages of in vitro cardiac differentiation from isolated MESP1-expressing-mesoderm progenitors. Sci. Rep..

[27] Lian X., Zhang J., Azarin S.M., Zhu K., Hazeltine L.B., Bao X., Hsiao C., Kamp T.J., Palecek S.P. (2013). Directed cardiomyocyte differentiation from human pluripotent stem cells by modulating Wnt/β-catenin signaling under fully defined conditions. Nat. Protoc..

[28] Nakanishi M., Kurisaki A., Hayashi Y., Warashina M., Ishiura S., Kusuda-Furue M., Asashima M. (2009). Directed induction of anterior and posterior primitive streak by Wnt from embryonic stem cells cultured in a chemically defined serum-free medium. FASEB J..

[29] Van den Ameele J., Tiberi L., Bondue A., Paulissen C., Herpoel A., Iacovino M., Kyba M., Blanpain C., Vanderhaeghen P. (2012). Eomesodermin induces Mesp1 expression and cardiac differentiation from embryonic stem cells in the absence of Activin. EMBO Rep..

[30] Bondue A., Blanpain C. (2010). Mesp1: A key regulator of cardiovascular lineage commitment. Circ. Res..

[31] Drowley L., Koonce C., Peel S., Jonebring A., Plowright A.T., Kattman S.J., Andersson H., Anson B., Swanson B.J., Wang Q.D. (2016). Human Induced Pluripotent Stem Cell-Derived Cardiac Progenitor Cells in Phenotypic Screening: A Transforming Growth Factor-beta Type 1 Receptor Kinase Inhibitor Induces Efficient Cardiac Differentiation. Stem Cells Transl. Med..

[32] Evseenko D., Zhu Y., Schenke-Layland K., Kuo J., Latour B., Ge S., Scholes J., Dravid G., Li X., MacLellan W.R. (2010). Mapping the first stages of mesoderm commitment during differentiation of human embryonic stem cells. Proc. Natl. Acad Sci. USA.

[33] Risebro C.A., Smart N., Dupays L., Breckenridge R., Mohun T.J., Riley P.R. (2006). Hand1 regulates cardiomyocyte proliferation versus differentiation in the developing heart. Development.

[34] Liu Y., Schwartz R.J. (2013). Transient Mesp1 expression: A driver of cardiac cell fate determination. Transcription.

[35] Yang Y.P., Li H.R., Cao X.M., Wang Q.X., Qiao C.J., Ya J. (2013). Second heart field and the development of the outflow tract in human embryonic heart. Dev. Growth Differ..

[36] Bizy A., Guerrero-Serna G., Hu B., Ponce-Balbuena D., Willis B.C., Zarzoso M., Ramirez R.J., Sener M.F., Mundada L.V., Klos M. (2013). Myosin light chain 2-based selection of human iPSC-derived early ventricular cardiac myocytes. Stem Cell Res..

[37] Franco D., Markman M.M., Wagenaar G.T., Ya J., Lamers W.H., Moorman A.F. (1999). Myosin light chain 2a and 2v identifies the embryonic outflow tract myocardium in the developing rodent heart. Anat. Rec..

[38] Saito Y., Nakamura K., Yoshida M., Sugiyama H., Ohe T., Kurokawa J., Furukawa T., Takano M., Nagase S., Morita H. (2015). Enhancement of Spontaneous Activity by HCN4 Overexpression in Mouse Embryonic Stem Cell-Derived Cardiomyocytes—A Possible Biological Pacemaker. PLoS ONE.

[39] Kubalak S.W., Miller-Hance W.C., O’Brien T.X., Dyson E., Chien K.R. (1994). Chamber specification of atrial myosin light chain-2 expression precedes septation during murine cardiogenesis. J. Biol. Chem..

[40] Skelton R.J., Costa M., Anderson D.J., Bruveris F., Finnin B.W., Koutsis K., Arasaratnam D., White A.J., Rafii A., Ng E.S. (2014). SIRPA, VCAM1 and CD34 identify discrete lineages during early human cardiovascular development. Stem Cell Res..

[41] Riedel M., Jou C.J., Lai S., Lux R.L., Moreno A.P., Spitzer K.W., Christians E., Tristani-Firouzi M., Benjamin I.J. (2014). Functional and Pharmacological Analysis of Cardiomyocytes Differentiated from Human Peripheral Blood Mononuclear-Derived Pluripotent Stem Cells. Stem Cell Rep..

[42] Fabiato A. (1983). Calcium-induced release of calcium from the cardiac sarcoplasmic reticulum. Am. J. Physiol..

[43] Chaudhari U., Nemade H., Wagh V., Gaspar J.A., Ellis J.K., Srinivasan S.P., Spitkovski D., Nguemo F., Louisse J., Bremer S. (2016). Identification of genomic biomarkers for anthracycline-induced cardiotoxicity in human iPSC-derived cardiomyocytes: An in vitro repeated exposure toxicity approach for safety assessment. Arch. Toxicol..

[44] Volkova M., Russell R. (2011). Anthracycline cardiotoxicity: Prevalence, pathogenesis and treatment. Curr. Cardiol. Rev..

[45] Boon E.M., Keller J.J., Wormhoudt T.A., Giardiello F.M., Offerhaus G.J., van der Neut R., Pals S.T. (2004). Sulindac targets nuclear beta-catenin accumulation and Wnt signalling in adenomas of patients with familial adenomatous polyposis and in human colorectal cancer cell lines. Br. J. Cancer.

[46] Lee H.-J., Wang N.X., Shi D.-L., Zheng J.J. (2009). Sulindac Inhibits Canonical Wnt Signaling by Blocking the PDZ Domain of Dishevelled. Angew. Chem. Int. Ed. Engl..

[47] Fuerer C., Nusse R. (2010). Lentiviral vectors to probe and manipulate the Wnt signaling pathway. PLoS ONE.

[48] Takahashi K., Tanabe K., Ohnuki M., Narita M., Ichisaka T., Tomoda K., Yamanaka S. (2007). Induction of pluripotent stem cells from adult human fibroblasts by defined factors. Cell.

[49] Cyranoski D. (2018). ‘Reprogrammed’ stem cells approved to mend human hearts for the first time. Nature.

[50] Martins A.M., Vunjak-Novakovic G., Reis R.L. (2014). The Current Status of iPS Cells in Cardiac Research and Their Potential for Tissue Engineering and Regenerative Medicine. Stem Cell Rev..

[51] Smith A.S., Macadangdang J., Leung W., Laflamme M.A., Kim D.H. (2016). Human iPSC-derived cardiomyocytes and tissue engineering strategies for disease modeling and drug screening. Biotechnol. Adv..

[52] Strober B.J., Elorbany R., Rhodes K., Krishnan N., Tayeb K., Battle A., Gilad Y. (2019). Dynamic genetic regulation of gene expression during cellular differentiation. Science.

[53] D’Antonio-Chronowska A., Donovan M.K.R., Young Greenwald W.W., Nguyen J.P., Fujita K., Hashem S., Matsui H., Soncin F., Parast M., Ward M.C. (2019). Association of Human iPSC Gene Signatures and X Chromosome Dosage with Two Distinct Cardiac Differentiation Trajectories. Stem Cell Rep..

[54] Kattman S.J., Witty A.D., Gagliardi M., Dubois N.C., Niapour M., Hotta A., Ellis J., Keller G. (2011). Stage-Specific Optimization of Activin/Nodal and BMP Signaling Promotes Cardiac Differentiation of Mouse and Human Pluripotent Stem Cell Lines. Cell Stem Cell.

[55] Wamstad J.A., Alexander J.M., Truty R.M., Shrikumar A., Li F., Eilertson K.E., Ding H., Wylie J.N., Pico A.R., Capra J.A. (2012). Dynamic and Coordinated Epigenetic Regulation of Developmental Transitions in the Cardiac Lineage. Cell.

[56] Zhang J., Klos M., Wilson G.F., Herman A.M., Lian X., Raval K.K., Barron M.R., Hou L., Soerens A.G., Yu J. (2012). Extracellular matrix promotes highly efficient cardiac differentiation of human pluripotent stem cells: The matrix sandwich method. Circ. Res..

[57] Cheng H., Lederer W.J., Cannell M.B. (1993). Calcium sparks: Elementary events underlying excitation-contraction coupling in heart muscle. Science.

[58] Guatimosim S., Guatimosim C., Song L.-S. (2011). Imaging Calcium Sparks in Cardiac Myocytes. Methods Mol. Biol..

[59] Itzhaki I., Rapoport S., Huber I., Mizrahi I., Zwi-Dantsis L., Arbel G., Schiller J., Gepstein L. (2011). Calcium handling in human induced pluripotent stem cell derived cardiomyocytes. PLoS ONE.

[60] Satin J., Itzhaki I., Rapoport S., Schroder E.A., Izu L., Arbel G., Beyar R., Balke C.W., Schiller J., Gepstein L. (2008). Calcium handling in human embryonic stem cell-derived cardiomyocytes. Stem Cells.

[61] Kempf H., Andree B., Zweigerdt R. (2016). Large-scale production of human pluripotent stem cell derived cardiomyocytes. Adv. Drug Deliv. Rev..

[62] Tohyama S., Fukuda K. (2017). Safe and Effective Cardiac Regenerative Therapy With Human-Induced Pluripotent Stem Cells. Circ. Res..

[63] Tohyama S., Hattori F., Sano M., Hishiki T., Nagahata Y., Matsuura T., Hashimoto H., Suzuki T., Yamashita H., Satoh Y. (2013). Distinct metabolic flow enables large-scale purification of mouse and human pluripotent stem cell-derived cardiomyocytes. Cell Stem Cell.

[64] Chaudhari U., Nemade H., Gaspar J.A., Hescheler J., Hengstler J.G., Sachinidis A. (2016). MicroRNAs as early toxicity signatures of doxorubicin in human-induced pluripotent stem cell-derived cardiomyocytes. Arch. Toxicol..

[65] Guo L., Qian J.Y., Abrams R., Tang H.M., Weiser T., Sanders M.J., Kolaja K.L. (2011). The electrophysiological effects of cardiac glycosides in human iPSC-derived cardiomyocytes and in guinea pig isolated hearts. Cell Physiol. Biochem..

[66] Kang J., Chen X.L., Ji J., Lei Q., Rampe D. (2012). Ca(2)(+) channel activators reveal differential L-type Ca(2)(+) channel pharmacology between native and stem cell-derived cardiomyocytes. J. Pharmacol. Exp. Ther..

[67] Muraoka N., Nara K., Tamura F., Kojima H., Yamakawa H., Sadahiro T., Miyamoto K., Isomi M., Haginiwa S., Tani H. (2019). Role of cyclooxygenase-2-mediated prostaglandin E2-prostaglandin E receptor 4 signaling in cardiac reprogramming. Nat Commun..

[68] Roos J., Grosch S., Werz O., Schroder P., Ziegler S., Fulda S., Paulus P., Urbschat A., Kuhn B., Maucher I. (2016). Regulation of tumorigenic Wnt signaling by cyclooxygenase-2, 5-lipoxygenase and their pharmacological inhibitors: A basis for novel drugs targeting cancer cells?. Pharm. Ther..

