# Correction: Nemade, H.; et al. Cyclooxygenases Inhibitors Efficiently Induce Cardiomyogenesis in Human Pluripotent Stem Cells. *Cells* 2020, *9*, 554

**DOI:** 10.3390/cells9102165

**Published:** 2020-09-24

**Authors:** Harshal Nemade, Aviseka Acharya, Umesh Chaudhari, Erastus Nembo, Filomain Nguemo, Nicole Riet, Hinrich Abken, Jürgen Hescheler, Symeon Papadopoulos, Agapios Sachinidis

**Affiliations:** 1 Institute of Neurophysiology, Faculty of Medicine, University of Cologne, Robert-Koch-Str. 39, 50931 Cologne, Germany; hnemade@uni-koeln.de (H.N.); aacharya@uni-koeln.de (A.A.); umeshchaudhari80@gmail.com (U.C.); nnembu@yahoo.com (E.N.); filo.nguemo@uni-koeln.de (F.N.); j.hescheler@uni-koeln.de (J.H.); symeon.papadopoulos@uk-koeln.de (S.P.); 2 Department I Internal Medicine and Center for Molecular Medicine Cologne (CMMC), University of Cologne (UKK), Robert-Koch-Str. 21, 50931 Cologne, Germany; nicole.riet@uk-koeln.de; 3 Regensburg Centre for Interventional Immunology (RCI), Department Genetic Immunotherapy, University Hospital Regensburg, 93053 Regensburg, Germany; Hinrich.Abken@klinik.uni-regensburg.de; 4 Center for Molecular Medicine Cologne (CMMC), University of Cologne, Robert-Koch-Str. 21, 50931 Cologne, Germany

The authors wish to make the following corrections to this paper [1]:

For the WNT reporter assay, the authors have taken six photos from each well for each sample. While making the final version of the figures for submission, the authors used two figures from the Sulindac group, which caused a duplication mistake in Figure 5a. 

The corrected Figure 5a is shown as follows:

**Figure 5:**
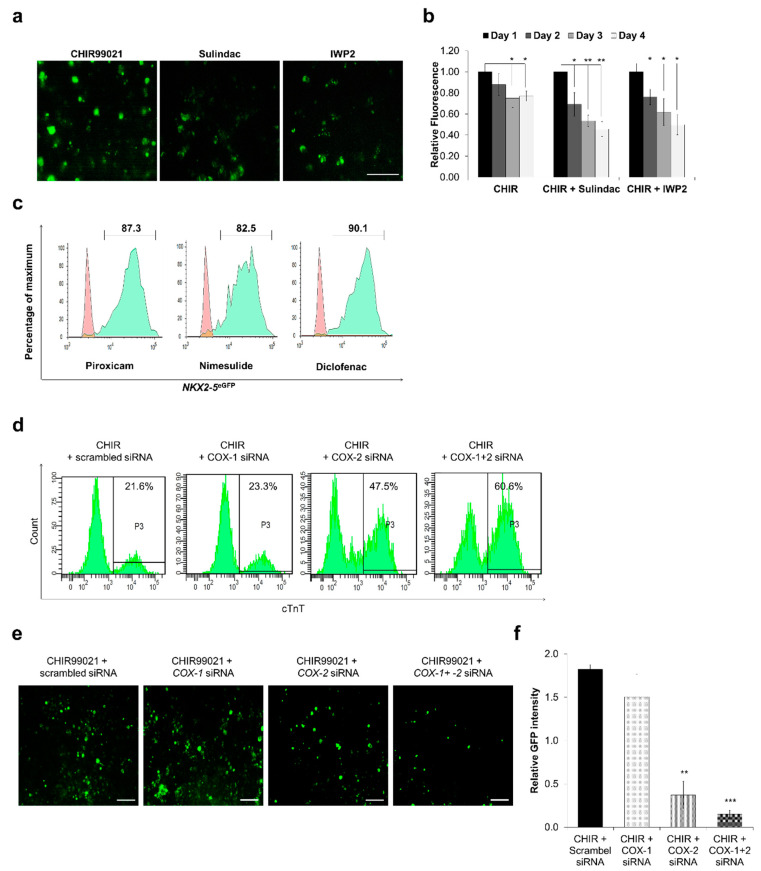
Cyclooxygenase inhibition plays an important role in cardiomyocytes differentiation. TCF reporter assay using IMR90-WNT reporter line: (**a**,**b**) The effect of Sulindac and IWP2 was examined. On day 0, CHIR99021 (10 μM) was added to activate Wnt signaling and TCF promoter activity from day 2 to day 4 cells were treated with Sulindac and IWP2. Fluorescence was recorded and images were captured on day 4. Scale bar, 100 μm (**a**), Error bars, ±SD; *n* = 3 independent biological replicates, (Student’s *t* test, * *p* ≤ 0.05, ** *p* ≤ 0.01, *** *p* ≤ 0.001) (**b**); (**c**) Representative FACS analyses for percent eGFP^+^ cells generated using Nimesulide, Diclofenac and Piroxicam. Control (red) indicates undifferentiated HES3 cells. (**d**) Representative FACS analyses for percent cTnT^+^ cells generated using COX-1, COX-2 and COX-1+2 siRNA. Undifferentiated IMR90 cells were used as control. (**e**,**f**) IMR90-WNT reporter cells were transfected with COX-1, COX-2 and COX-1+2 siRNAs after treatment with CHIR99021. Fluorescence images were captured on day 4 and analysed using ImageJ to obtain relative GFP intensities. Scale bar = 50 μm, Error bars, ±SEM; *n* = 3 independent biological replicates, (Student’s *t* test, * *p* ≤ 0.05, ** *p* ≤ 0.01, *** *p* ≤ 0.001).

The authors would like to apologize for any inconvenience caused to the readers by these changes. These corrections do not affect the study’s results or conclusions. 
