# Supplementary material for: Cyclooxygenases Inhibitors Efficiently Induce Cardiomyogenesis in Human Pluripotent Stem Cells
Source: Cells. 2020 Feb 27;9(3):554. doi: 10.3390/cells9030554 (PMC7140528; doi:10.3390/cells9030554)
Supplement: Supplementary file 1 [file cells-09-00554-s001.zip › SM-revise/cells-699813-SM revise.pdf]

Supplementary Figure 1

S1A

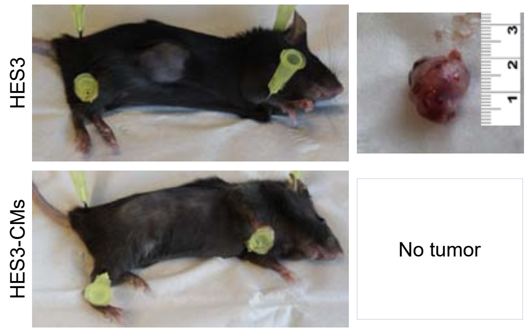

S1B

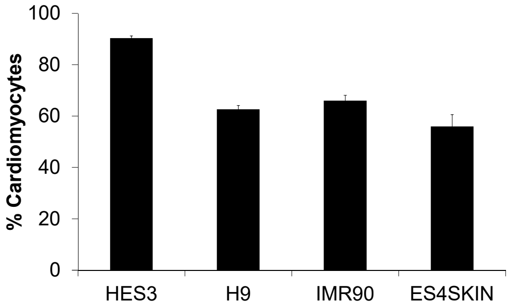

Figure S1. Tumorigenicity of Sulindac-derived HES3-CMs and applicability of Sulindac-based cardiac differentiation protocol.
(A) Immunosuppressed Rag$^{-/-}$ mice were injected with undifferentiated HES3 cells (Group-1: 4 mice, 3×10^5 cells/mice), purified Sulindac-derived HES3-CMs (Group-2: 4 mice, 3×10^5 cells/mice) and PBS (Control: 2 mice). Visible tumours were observed in Group-1 mice after 3 weeks whereas no tumours were detected in Group-2 and Control mice populations. (B) Cardiogenic effect of Sulindac on hPSC lines. hESCs (HES3 and H9) and hiPSCs (IMR90 and ES4SKIN) were differentiated into cardiomyocytes by treatment with 10 μM Sulindac. At day 12 cells were analyzed for cTnT expression using flow cytometry. Error bars, ±SEM; n = 3 independent biological replicates.

Supplementary Figure 2

S2A

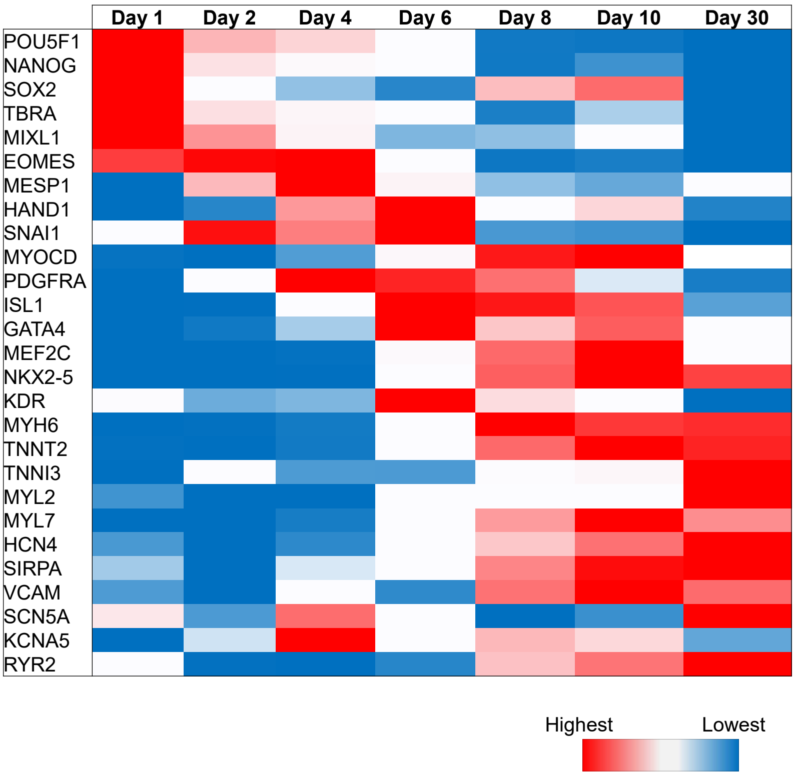

S2B

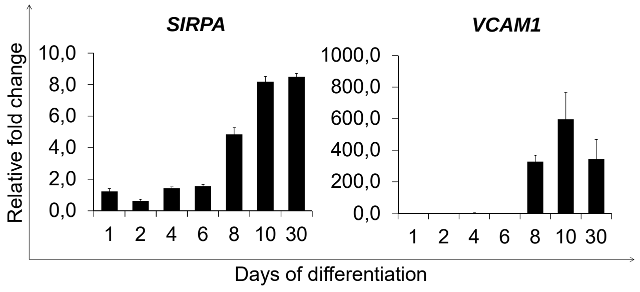

S2C

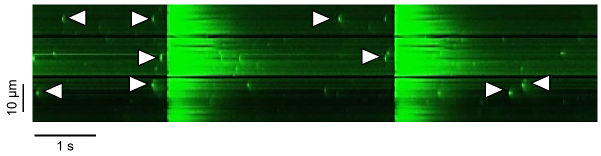

S2D

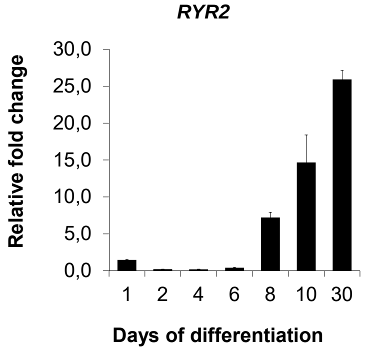

Figure S2. Differentiation and characterization of Sulindac-derived HES3-CMs.
(A) Heat map of mRNA expression profiles for 27 target genes analyzed using qRT-PCR during Sulindac-based cardiac differentiation protocol. Target genes include markers for pluripotency, early mesoderm, MPCs, CPCs and CMs. (B) Gene expression analysis by qRT-PCR for cardiomyocyte specific surface markers *SIRPA* and *VCAM1*. Error bars, ±SEM; n = 3 independent biological replicates. (C) Line scan images recorded from Sulindac-derives HES3-CMs of spontaneous Ca^{2+} transients with calcium sparks (Ca^{2+} sparks-white arrow heads). (D) Gene expression analysis by qRT-PCR for cardiac ryanodine receptor (*RYR2*). Error bars, ±SEM; n = 3 independent biological replicates.

Supplementary Figure 3

S3A

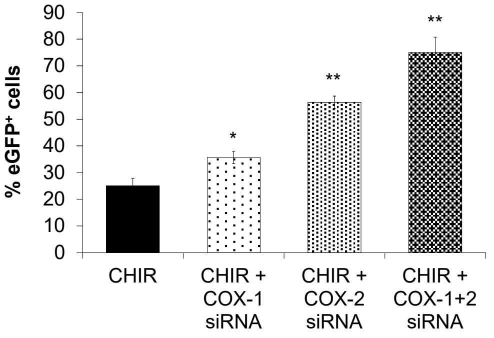

Figure S3. Differentiation of HES3 cells using COX siRNA.
(A) HES3 cells were transfected with COX-1, COX-2 and COX-1+2 siRNAs and %eGFP$^+$ cells were measured on day 12 using FACS. Error bars, ±SEM; n = 3 independent biological replicates, Student's t test $*p < 0.05$, $**p < 0.01$.

Supplementary Tables

Table S1

APs (n=29)	MDP (mV)	Height (mV)	Frequency (bpm)	dV/dt max (V/s)	APD10 (ms)	APD50 (ms)	APD90 (ms)
**Ventricular-like (n=17)**	-54.93±0.52	108.75±0.48	72.19±1.56	20.67±0.46	107.32±1.30*	209.91±2.17*	439.09±10.41*
**Atrial-like (n=7)**	-50.80±0.47	85.60±0.81	82.26±5.42	8.22±0.23	45.13±1.23*	121.85±2.18*	275.28±8.76*
**Nodal-like (n=5)**	-29.21±1.32	60.10±0.78	157.80±8.13	3.40±0.098	40.50±2.45*	93.10±2.25*	200.90±9.11*

Table S2

	Expt.1	Expt.2	Expt.3	Total number of cells	Cell population (%)
Ventricular	24	10	17	51	62.9
Atrial	8	5	7	20	24.7
Nodal	4	1	5	10	12.3
Total	36	16	29	81	100

Supplementary Table. Electrophysiological characterization of Sulindac-derived HES3-CMs. Table S1) AP parameters for the three CM subtypes. MDP = maximum diastolic potential, dV/dt max = maximum rate of rise of AP, APD10/APD/50/APD90 = AP duration measured at 10%, 50% or 90% of repolarization. Values are mean ± SEM. *p < 0.05 (ANOVA with Bonferroni correction). Table S2) Based on the AP characteristics like APA (action potential amplitude), action potential duration (APD) and dV/dtmax CMs were classified into atrial-, nodal-, or ventricular-like CMs. Table represents percent population of each three CM cell sub-types in CM population. (n = 3).

Table S3

	F0	(ΔF/ΔT) max	Fmax /F0	TTP	FWFM	T90%
HES3-CMs	467.58 ±45.89	164.06 ±20.69	2.32 ±0.14	131.43 ±20.43	599.98 ±57.91	778.32 ±74.16

Supplementary Table S3. Calcium transient properties of Sulindac-derived HES3-CMs. Table represents Ca^{2+} transient parameters (in msec) measured from Sulindac-derived HES3-CMs loaded with Cal-520AM Ca^{2+} indicator. F/F0, Ca^{2+} transient amplitude where F0 is the averaged background-corrected resting fluorescence intensity; TTP, time-to-peak; T90%, 90% recovery of Fmax; [ΔF/ΔT]max, the maximum steepness; FWHM, full width at half maximum. (n = 25).

Supplementary Methods

Immunocytochemistry
Antibodies against the following proteins were used in this study: Oct3/4 (SC5279, Santa Cruz), Nanog (SC33759, Santa Cruz), SSEA-4 (SC21704, Santa Cruz), Sarcomeric Alpha Actinin (α-actinin) (ab9465, Abcam), Cardiac Troponin T (cTnT) (ab45932, Abcam) and RYR2 (HPA020028, Atlas Antibodies). The HES3-CMs were fixed with 4% paraformaldehyde (PFA), permeabilized with 0.3 % Triton X-100 and blocked with 5% Bovine Serum Albumin (BSA) at room temperature. Then the cells were incubated with primary antibody in blocking solution. Subsequently, cells were incubated with the appropriate secondary antibodies conjugated with Alexa-Fluor 488 or 594 (A32723 or R37117, ThermoFisher) or FITC (SC2012 or SC2080, Santa Cruz). The cells were then washed and mounted with Prolong® Gold anti-fade mount with DAPI (P36931, ThermoFisher). The images were acquired with an Axiovert 200 fluorescence microscope and Axiovision 4.3 software (Carl Zeiss).

Flow Cytometry
Beating clusters were dissociated into single cells using StemPro® Accutase® Cell Dissociation Reagent (Thermo Fisher, MA, US) and were then fixed with ice-cold 99% Methanol for 10 min at room temperature and stained with anti-cTnT antibody (Abcam) in DPBS plus 0.1% Triton X-100 and 0.5% BSA. In case of HES3-CMs, to quantify the eGFP+ population, the cells were dissociated and fixed with ice-cold 99% Methanol. Flow cytometric data were collected and analyzed using Attune® Acoustic Focusing Cytometer and Attune® Cytometric Software v2.1 (Applied Biosystems, CA, US) respectively.

Electron Microscopy
For TEM study, HES3-CMs were seeded onto fibronectin-coated ACLAR® embedding film (Ted Pella, CA, US) in 96-well plate. 48 h post seeding, cells were washed with DPBS (+/+) and fixed using 2% glutaraldehyde and 2% formaldehyde in 0.1 M cacodylate buffer (pH 7.3) for 2 h at 4°C, post fixed in 1% OsO4 for 30 min at 4°C in dark. After washing, cell samples were dehydrated in graded ethanol and embedded into Epon (20 g Epoxy, 11 g DDSA, 9 g NMA, 0.8 g DMP-30) (Sigma-Aldrich) for 48 to 72 h at 62°C. Ultrathin (~70 nm) sections were obtained using Leica EM UC6 Ultramicrotome (Leica, Germany) and double-stained with uranyl acetate followed by lead citrate. Ultrastructural analysis was done using a transmission electron microscope (EM109, Zeiss, Germany) equipped with slow-scan CCD-camera (TRS).

The xCELLigence RTCA Cardio system
The xCELLigence RTCA Cardio system (ACEA Biosciences, San Diego, CA, USA) is an impedance-based platform for monitoring the real-time beating function of cardiomyocytes. The E-plate Cardio 96 (ACEA Biosciences, San Diego, CA, USA) xCELLigence plates were equilibrated by using iCell-PM (50 µl per well) and inserted into the xCELLigence station to measure background impedance and to ensure that all wells and connections were working within acceptable limits. After equilibration the HES3-CMs were harvested and seeded in required density. Impedance measurements were monitored at regular time intervals. The amount of growth area covered in an E-plate Cardio 96 due to cell adhesion was represented as the Cell Index (CI). A high CI indicates more cell adhesion and vice versa. The raw data and statistical information, such as mean and SD for the parameters like CI, beating rate, amplitude, normalized CI, normalized beating rate, and normalized amplitude were acquired using RTCA Cardio software version 1.0 (ACEA Biosciences, Inc., San Diego, CA, USA).

RNA isolation and Quantitative RT-PCR
To analyze the mRNA expression, cells were homogenized with QIAzol lysis reagent (QIAGEN, Germany), and the total RNA was extracted using the miRNeasy Mini Kit (QIAGEN, Germany) according to the manufacturer's instructions. Extracted RNA was assessed for purity and quantity using Nanodrop (ND-1000, Thermo Fisher, Germany). 500 ng of total RNA was subjected to cDNA synthesis using RT2 First Strand kit (QIAGEN, Germany) according to the manufacturer's instructions. Quantitative comparison of mRNA levels was performed in triplicates using custom made RT2 Profiler PCR array (96-well plate) (QIAGEN, Germany) containing 27 target genes, 2 housekeeping genes, 1 genomic DNA control, 1 reverse transcription controls and 1 positive PCR controls. The qRT-PCR was performed using RT2 SYBR® Green ROX™ qPCR master mix (QIAGEN, Germany) in an Applied Biosystems 7500 FAST Real-Time PCR System in accordance with the manufacturer's recommended thermal cycling conditions. The relative gene expression analysis was performed using the 2−ΔΔCt method (Dardousis et al. 2007). Fold change values were normalized with respect to GAPDH expression level and expressed relative to the corresponding values in day 0 control sample. All of the 27 target genes are listed in Figure S3A.

Action potential measurements
For action potential (AP) recordings, day 30 HES3-CMs were dissociated into single cells and plated on sterile 0.1% gelatin-coated glass coverslip. 48 h post plating, APs were recorded by the whole-cell current-clamp technique using PULSE program and EPC 9 amplifier (HEKA) (Nembo et al. 2015). Briefly, patch pipettes with resistance of 2–3 MΩ were prepared from glass capillary tubes (Harvard Apparatus Ltd, UK) using a two-stage horizontal puller (DMZ Universal Puller, Germany) and filled with intracellular solution containing (in mM): 50 KCl, 80 potassium L-aspartate, 1 MgCl2, 10 EGTA, 3 Mg-ATP, and 10 Hepes (pH 7.2; KOH). Glass coverslip containing HES3-CMs then placed into a temperature-controlled recording chamber (37°C) and continuously perfused with the standard extracellular solution containing (in mM): 140 NaCl, 5.4 KCl, 1.8 CaCl2, 1 MgCl2, 10 Hepes, 10 glucose (pH 7.4; NaOH). Pulse (HEKA, Germany) was used for data acquisition whereas Pulse Fit and AP analysis were used for data analysis.

Teratoma Analysis
The teratoma assay was performed on SCID (Rag2−/−common gamma−/−) mice, as described previously (Faitschuk et al. 2016). The animal experiments have been approved by the Universitätsklinikum Köln (Institutional Ethics Review Board reference number 01-090) and the governmental animal care and use office (Landesamt für Natur, Umwelt und Verbraucherschutz Nordrhein- Westfalen, Recklinghausen, Germany (reference number 84-02.04.2012.A417 LANUV). Briefly, HES3 ESCs (4 mice, 3 × 105 cells/mice), purified Sulindac-derived HES3-CMs (4 mice, 3 × 105 cells/mice) and PBS (2 mice) in 200 µL DMEM were injected subcutaneously into the dorsal flanks of SCID (Rag2−/−common gamma−/−) mice. At 3 weeks after these injections, the tumor formation has been checked.

Supplementary References

Dardousis K, Voolstra C, Roengvoraphoj M, et al. (2007) Identification of differentially expressed genes involved in the formation of multicellular tumor spheroids by HT-29 colon carcinoma cells. Mol Ther 15(1):94-102 doi:10.1038/sj.mt.6300003

Faitschuk E, Hombach AA, Frenzel LP, Wendtner CM, Abken H (2016) Chimeric antigen receptor T cells targeting Fc mu receptor selectively eliminate CLL cells while sparing healthy B cells. Blood 128(13):1711-22 doi:10.1182/blood-2016-01-692046

Nembo EN, Atsamo AD, Nguelefack TB, Kamanyi A, Hescheler J, Nguemo F (2015) In vitro chronotropic effects of Erythrina senegalensis DC (Fabaceae) aqueous extract on mouse heart slice and pluripotent stem cell-derived cardiomyocytes. J Ethnopharmacol 165:163-72 doi:10.1016/j.jep.2015.02.002
